# ﻿A new species of *Pseudopoda* (Araneae, Sparassidae) from China, with first description of the female of *P. spiralis* Zhang, Jäger & Liu, 2023

**DOI:** 10.3897/zookeys.1260.158762

**Published:** 2025-11-18

**Authors:** He Zhang, Chang-Hao Hu, Lu-Yu Wang, Jie Liu, Yang Zhong

**Affiliations:** 1 School of Nuclear Technology and Chemistry & Biology, Hubei University of Science and Technology, Xianning, Hubei, China; 2 College of Physics and Electronic Engineering, Xingtai University, Xingtai, Hebei, China; 3 Hebei Provincial Sweet Potato Breeding and Application Technology Innovation Center, Xingtai, Hebei, China; 4 Arachnid Resource Centre of Hubei Province & Hubei Key Laboratory of Regional Development and Environmental Response, Faculty of Resources and Environmental Science, Hubei University, Wuhan, Hubei, China; 5 Centre for Behavioral Ecology and Evolution, School of Life Sciences, Hubei University, Wuhan, Hubei, China; 6 College of Life Sciences, Hunan Normal University, Changsha, Hunan, China; 7 Key Laboratory of Eco-environments in Three Gorges Reservoir Region (Ministry of Education), School of Life Sciences, Southwest University, Chongqing, China

**Keywords:** Biodiversity, Heteropodinae, huntsman spiders, morphology, taxonomy

## Abstract

One new species of *Pseudopoda* Jäger, 2000 is described from Guangxi, southern China: *P. rongxianensis***sp. nov.** Additional materials of *P. deformis* Gong & Zhong, 2023 and *P. spiralis* Zhang, Jäger & Liu, 2023 are also presented, with the female of *P. spiralis* described for the first time. Figures and distribution data of these three species are provided.

## ﻿Introduction

The genus *Pseudopoda* Jäger, 2000 is currently the most species-rich genus within the family Sparassidae Bertkau, 1872, comprising 270 valid species from East, South, and Southeast Asia (World Spider Catalog 2025). These spiders are typically found in forested and montane ecosystems, often inhabiting leaf litter, vegetation and under rocks in humid environments (Jäger 2015; Zhang et al. 2023). Across its distribution, China represents the most significant center of diversity for the genus, with over 160 species recorded to date. This high number of species, along with the frequent discovery of new taxa, suggests considerable endemism and an underestimation of the true species richness (Zhang et al. 2023).

Despite ongoing taxonomic efforts, the classification and understanding of *Pseudopoda* remain incomplete. Of the known species, only 68 have been assigned to defined species groups based on morphological or integrative evidence, including the *daliensis*-group, *diversipunctata*-group, *interposita*-group, *latembola*-group, *martensi*-group, *parvipunctata*-group, *prompta*-group, *schwendingeri*-group, and *signata*-group (Jäger 2001; Zhang et al. 2017, 2019; Li et al. 2019; Zhang et al. 2025). The remaining 202 species are still ungrouped, highlighting the complexity and unresolved relationships within the genus. Moreover, 138 species are currently known only from a single sex, which poses ongoing challenges to accurate species identification and comparative morphological analyses (Wu et al. 2024).

In this study, we describe one new species of *Pseudopoda* collected from Guangxi Zhuang Autonomous Region, China. We also present new morphological information and a distributional record of *Pseudopoda deformis* Gong & Zhong, 2023 (Gong et al. 2023), based on a female specimen from Chongqing Municipality, which documents notable intraspecific variation in the internal duct system. In addition, we describe the female of *Pseudopoda spiralis* Zhang, Jäger & Liu, 2023 (Zhang et al. 2023) for the first time. These findings contribute to a more complete understanding of the genus’s diversity and distribution in China.

## ﻿Material and methods

All specimens were collected by hand and examined using an Olympus SZX16 stereomicroscope; further details were investigated using an Olympus BX51 compound microscope. Colouration is described in all species from specimens in ethanol. Copulatory organs were examined and illustrated after dissection from the spider bodies; epigynes were cleared with Proteinase K. Habitus photos were obtained using a Leica M205C digital microscope attached with a Leica DMC4500 digital camera.

Leg measurements are shown as: total length (femur, patella, tibia, metatarsus, tarsus). The number of spines is listed for each segment in the following order: prolateral, dorsal, retrolateral, ventral (in femora and patellae ventral spines are absent, and the fourth digit is omitted in the spination formula). The terminology used in text and figure legends follows Jäger (2000). All measurements are given in millimetres. The classes of somatic size follow the criteria of Jäger (2001). Arising points of the tegular appendages of male palps are given according to clock positions of the left palp in ventral view (Rheims 2013). The map was produced using ArcMap version 10.8.1.

Abbreviations used in text and figures: **ALE**, anterior lateral eyes; **AME**, anterior median eyes; **C**, conductor; **CH**, clypeus height; **CO**, copulatory opening; **CP**, cymbial process; **dRTA**, dorsal branch of RTA; **DS**, dorsal shield of prosoma; **E**, embolus; **EP**, embolic projection; **FD**, fertilization duct; **Fe**, femur; **FW**, first winding; **IDS**, internal duct system; **LL**, lateral lobes; **Mt**, metatarsus; **OS**, opisthosoma; **Pa**, patella; **PLE**, posterior lateral eyes; **PME**, posterior median eyes; **Pp**, palp; **RTA**, retrolateral tibial apophysis; **Sp**, spermophor; **St**, subtegulum; **T**, tegulum; **Ti**, tibia; **vRTA**, ventral branch of RTA; **VTA**, ventral tibial apophysis; **I**, **II**, **III**, **IV**, legs I to IV.

The studied specimens have been deposited in the Centre for Behavioral Ecology and Evolution, College of Life Sciences, Hubei University, Wuhan, China (**CBEE**), and the School of Nuclear Technology and Chemistry and Biology, Hubei University of Science and Technology, Xianning, China (**HUST**).

## ﻿Taxonomy

**Family Sparassidae Bertkau, 1872**

**Subfamily Heteropodinae Thorell, 1873**

### ﻿Genus Pseudopoda

Taxon classificationAnimaliaAraneaeSparassidae

Jäger, 2000

C357048B-576E-5B86-8E06-CD6EE39D547E

#### Type species.

*Pseudopoda prompta* (O. Pickard-Cambridge, 1885).

#### Diagnosis.

See Jäger (2000).

#### Distribution.

East, South and Southeast Asia.

### ﻿ Pseudopoda rongxianensis sp. nov.

Taxon classificationAnimaliaAraneaeSparassidae

1C4B6F06-5BC8-57B4-8FD0-7D18F6DA2E84

https://zoobank.org/C55D09FD-D7BA-41F8-B3AF-60ACEF0E7027

Figs 1, 2, 8

#### Type material.

**Holotype** male: **China • Guangxi Zhuang Autonomous Region**: Yulin City, Rong County, Duqiaoshan Forest Park, 22.8216°N, 110.6429°E, 187 m, 5 October 2023, Qian-Le Lu leg. (CBEE, GXRX-23-1001).

#### Etymology.

The specific name is derived from the type locality, Rong County.

#### Diagnosis.

**Figure 1.**
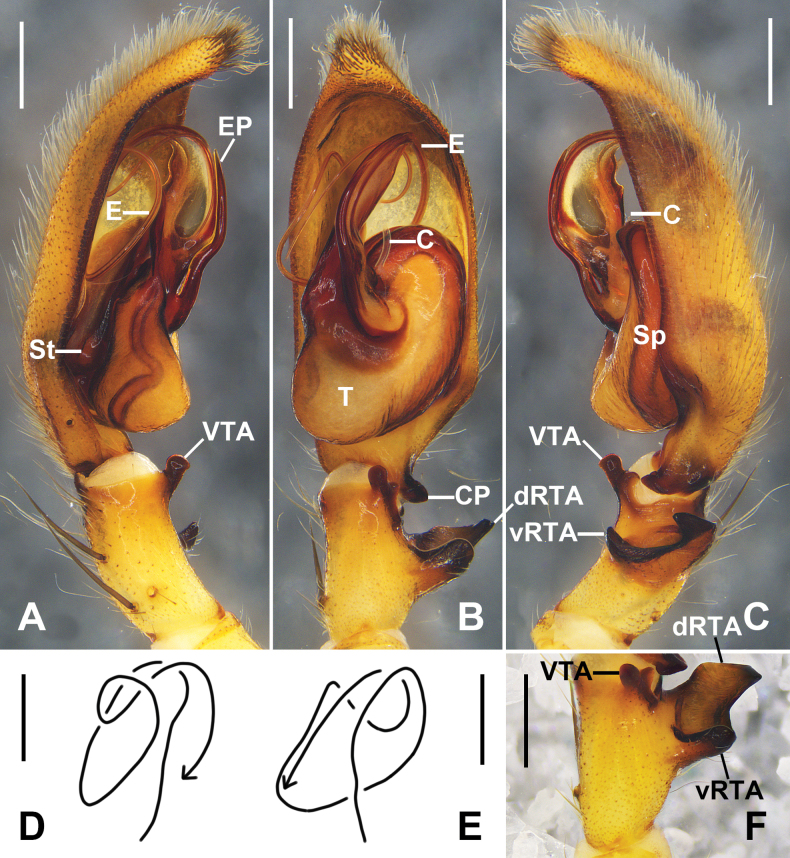
*
Pseudopoda rongxianensis* sp. nov. from Guangxi, left palp of male holotype (A–C). A. Ventral; B. Dorsal; C. Retrolateral; D. Schematic course of embolus, prolateral view; E. Schematic course of embolus, ventral view; F. Left male palpal tibia, retrolateral. Abbreviations: C, conductor; CP, cymbial process; dRTA, dorsal branch of RTA; E, embolus; EP, embolic projection; Sp, spermophor; St, subtegulum; T, tegulum; vRTA, ventral branch of RTA; VTA ventral tibial apophysis. Scale bars: 0.5 mm.

Male of *P. rongxianensis* sp. nov. is similar to that of *P. strombuliformis* Zhang, Jäger & Liu, 2023 (cf. Fig. 1 and Zhang et al. 2023: fig. 231) in having large alveolus, and an extremely elongated and filiform embolus, but can be distinguished from *P. strombuliformis* by: 1) vRTA longer than the connecting part between vRTA and dRTA; 2) VTA thinner, almost 1/4 of the width of tibia; 3) prolateral part of tegulum without a depression; 4) embolic projection long, almost as long as the diameter of the first loop of embolus; and 5) embolus long, forming 3 loops (vs. vRTA shorter than the connecting part between vRTA and dRTA, VTA almost 1/3 of the width of tibia, prolateral tegulum with depression, embolic projection shorter, almost half the length of the diameter of the first loop of embolus, embolus forming 7 loops in *P. strombuliformis*).

#### Description.

**Male (holotype)**: Small-sized. Body length 8.9, DS length 4.0, width 3.5, OS length 4.3, width 2.9. Eye measurements: AME 0.18; ALE 0.32; PME 0.25; PLE 0.31; AME–AME 0.11; AME–ALE 0.04; PME–PME 0.16; PME–PLE 0.28; AME–PME 0.24; ALE–PLE 0.13; CH AME 0.28; CH ALE 0.28. Measurements of palp and legs: Pp: 6.5 (2.0, 0.7, 1.1, 2.7); I 19.0 (5.3, 1.5, 5.8, 4.8, 1.6); II 20.7 (5.9, 1.6, 6.2, 5.2, 1.8); III 15.7 (4.6, 1.4, 4.4, 3.9, 1.4); IV 18.8 (5.6, 1.3, 5.0, 5.2, 1.7). Spination: Pp: 131, 101, 2111; legs: Fe I–II 323, III 322, IV 321; Pa I–IV 001; Ti I –II 2026, III–IV 2126; Mt I–II 2024, III 3036, IV 3236. Leg formula: II-I-IV-III. Chelicerae with 3 promarginal, 4 retromarginal teeth, and c. 16 denticles.

***Palp*** (Fig. 1A–F): As in diagnosis. Tibia almost 1/3 of the length of cymbium, RTA arising medially from tibia, vRTA with rounded distal part in ventral view, dRTA quadrangular in ventral view, ventral distal tibia with a short blunt apophysis. Retroproximal cymbium with a short blunt process (paracymbium in Ramírez 2014); alveolus extending into distal cymbial half. Conductor filiform, arising from tegulum at 11 o’clock position. Embolic projection slightly curved, almost 2/5 of the length of cymbium. Embolus arising sub-centrally from tegulum.

**Figure 2.**
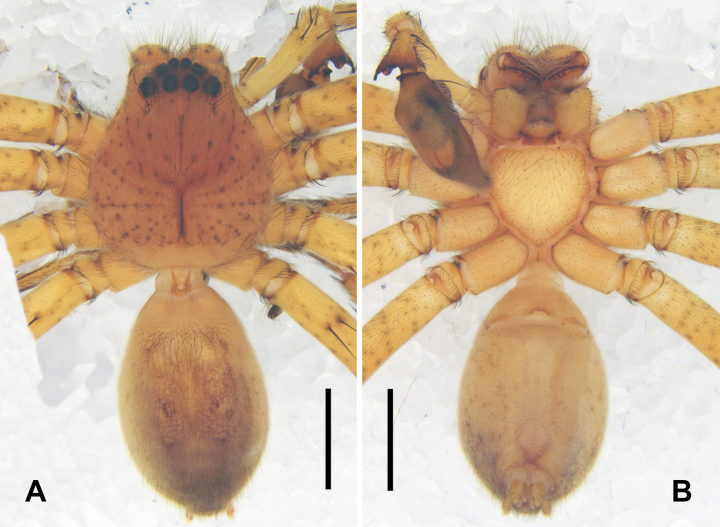
*
Pseudopoda rongxianensis* sp. nov. from Guangxi, male habitus. A. Dorsal; B. Ventral. Scale bars: 2 mm.

***Colouration*** (Fig. 2A, B): Carapace light orange, with black spots except posterior margin. Chelicerae light orange, with black spots. Legs yellow, with black spots. Sternum yellow, with orange margins. Opisthosoma yellowish-grey, dorsum medially with four light brown dots, venter laterally with light brown spots, in front of spinnerets with a light brown V-shaped marking.

**Female**: unknown.

#### Remarks.

This species shows similar palpal morphology to *Pseudopoda strombuliformis* (e.g., large alveolus, extremely elongated and filiform embolus, and short and quadrangular dRTA), differing from other congeners. These two species probably belong to an undefined species group. The stick-shaped embolus projection, and long and filiform embolus are similar to *Martensopoda* Jäger, 2006, a genus endemic to the southern mountain ranges of India (Jäger 2006). Therefore, we considered that these two *Pseudopoda* species may represent a transitional group between *Pseudopoda* and *Martensopoda*, or that the morphological similarities may result from convergent evolution.

#### Distribution.

China (Guangxi Zhuang Autonomous Region, Fig. 8).

### ﻿ Pseudopoda deformis

Taxon classificationAnimaliaAraneaeSparassidae

Gong & Zhong, 2023

7015A467-FEDB-5A63-A882-A997D3EC6B62

Figs 3, 4, 8

Pseudopoda deformis Gong & Zhong, in Gong et al. 2023: 191, figs 1A–F, 2A–F, 3A–J (male holotype, male and female paratypes from Guanmenshan Scenic Area, Muyu Town, Shennongjia Forestry District, Hubei Province, China, deposited in HUST, examined).

#### Other material examined.

**China** • Chongqing Municipality: 1 female, Wuxi County, Shuangyang Township, Yintiaoling Nature Reserve, Linkouzi, 31.4746°N, 109.8778°E, 1224 m, 22 June 2024, Luyu Wang leg. (CBEE, LJ20240053).

#### Diagnosis.

See Gong et al. (2023). The additional female examined has more compact and medially shifted copulatory ducts, consistent with intraspecific variation.

#### Description.

**Figure 3.**
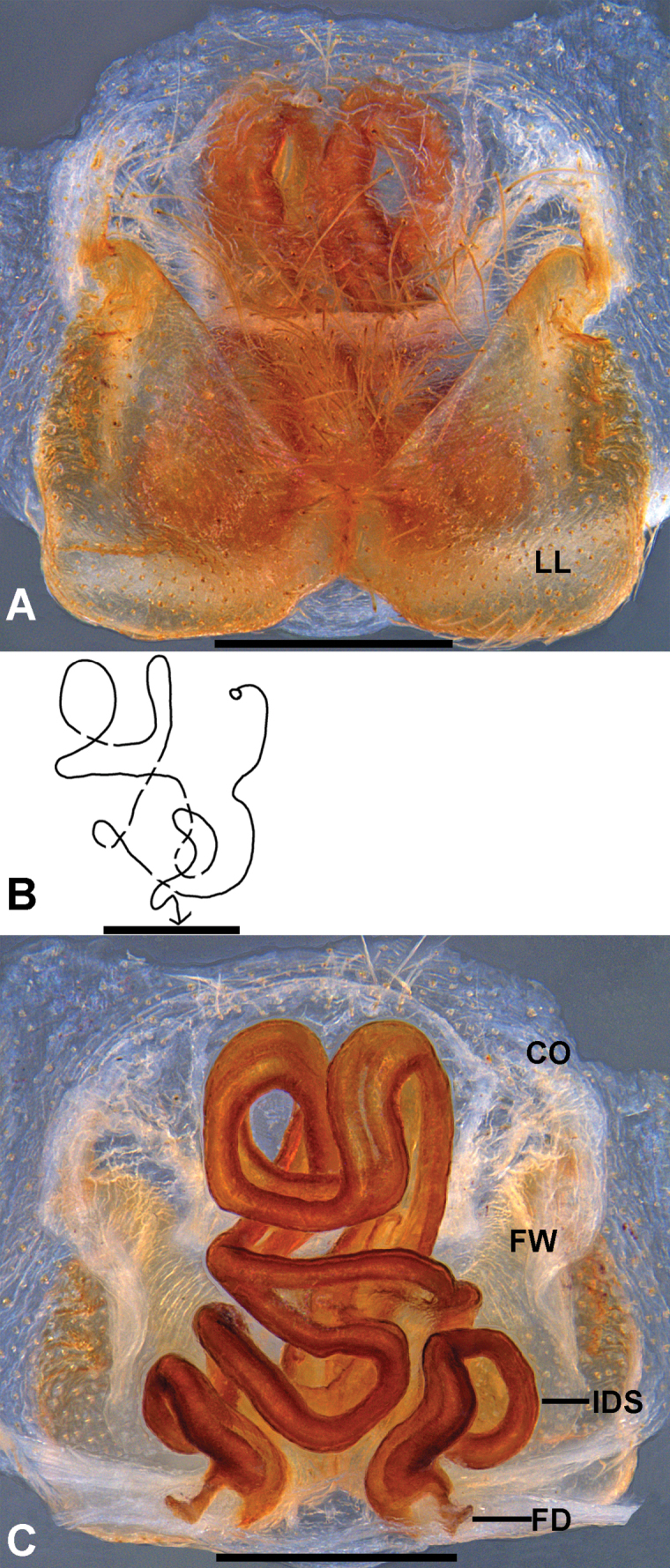
*
Pseudopoda deformis* Gong & Zhong, 2023 from Chongqing, female. A. Epigyne, ventral; B. Schematic course of IDS, dorsal; C. Vulva, dorsal. Abbreviations: CO, copulatory opening; FD, fertilization duct; FW, first winding; IDS, Internal duct system; LL, lateral lobes. Scale bars: 0.5 mm.

**Figure 4.**
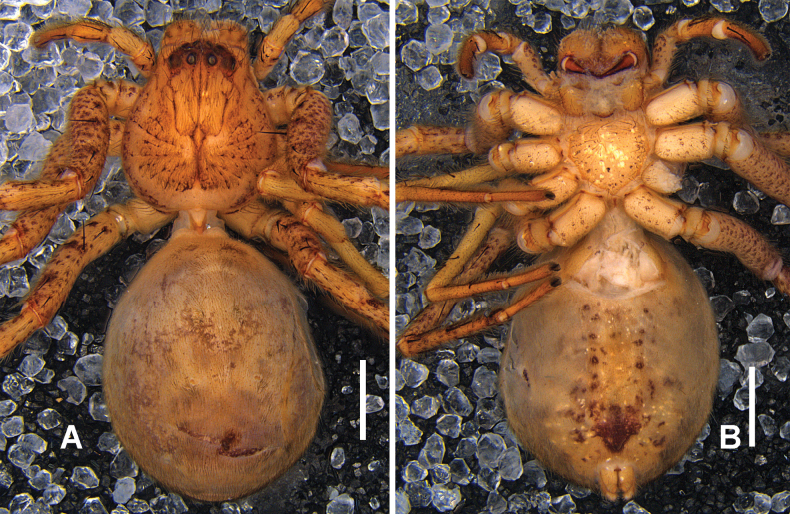
*
Pseudopoda deformis* Gong & Zhong, 2023 from Chongqing, female habitus. A. Dorsal; B. Ventral. Scale bars: 2 mm.

Female (Figs 3, 4): Internal duct system more compactly coiled, FD more twisted, medially shifted (Fig. 3A, B). For further description, see Gong et al. (2023).

#### Remarks.

All examined specimens are considered conspecific based on the general structure and configuration of the epigynal field and vulval organs. The internal duct system is highly convoluted and exhibits no consistent orientation or symmetry, reflecting a high degree of structural complexity and notable intraspecific variation.

#### Distribution.

China (Hubei Province and Chongqing Municipality, Fig. 8).

### ﻿ Pseudopoda spiralis

Taxon classificationAnimaliaAraneaeSparassidae

Zhang, Jäger & Liu, 2023

65140E7D-445E-5BAB-A343-87FDB0EDCD62

Figs 5, 6, 7, 8

Pseudopoda spiralis Zhang, Jäger & Liu, 2023 in Zhang et al. 2023: 247, figs 229A–C, 230A–B (male holotype from Maoxian Mountain Ecosystem Positioning Station, CAS, Sichuan, China, deposited in CBEE, examined).

#### Other material examined.

**China** • Sichuan Province: 4 males, 4 females, with same data as for holotype, except for: 25–26 May 2015, Gui-Qiang Huang, Lu-Yu Wang & Yan-Chen Zhou leg. (CBEE, LJ20240001–LJ20240003, LJ20240007, LJ20240015, LJ20240096–LJ20240098).

#### Diagnosis.

The female of this species can be distinguished from congeners by its internal duct system, which is elongate, narrow, and forms a compact, longitudinally aligned looping pattern, clearly visible in dorsal view.

#### Description.

**Figure 5.**
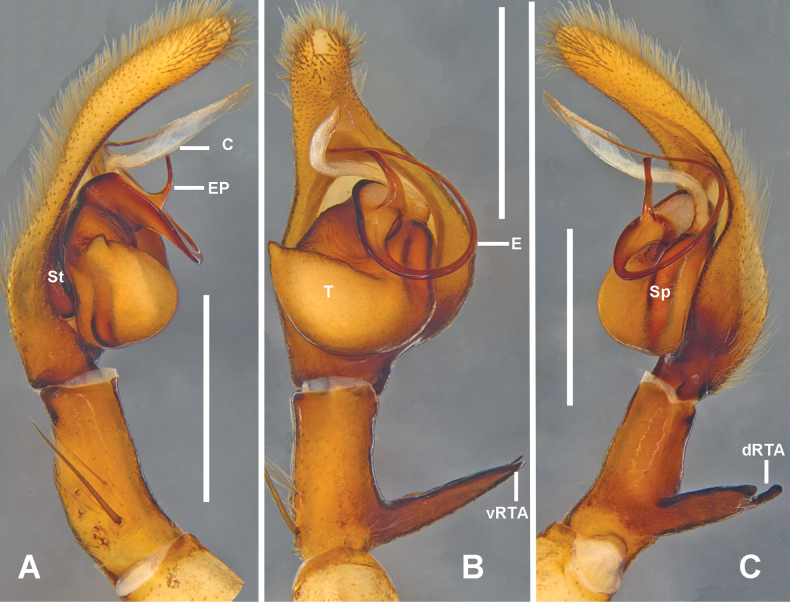
*
Pseudopoda spiralis* Zhang, Jäger & Liu, 2023 from Sichuan, left male palp. A. Ventral; B. Dorsal; C. Retrolateral. Abbreviations: C, conductor; dRTA, dorsal branch of RTA; E, embolus; EP, embolic projection; Sp, spermophor; St, subtegulum; T, tegulum; vRTA, ventral branch of RTA. Scale bars: 1 mm.

**Male** (Figs 5, 7A, B): for details see Zhang et al. (2023).

**Female** (LJ20240007): Measurements: Small-sized. Body length 8.2, DS length 3.3, width 3.1, OS length 4.9, width 3.7. Eyes: AME 0.13, ALE 0.20, PME 0.16, PLE 0.18, AME–AME 0.17, AME–ALE 0.07, PME–PME 0.21, PME–PLE 0.32, AME–PME 0.24, ALE–PLE 0.22, CH AME 0.36, CH ALE 0.30. Spination: Pp 131, 101, 2121, 1014; Fe I–II 323, III 322, IV 321; Pa I–III 101, IV 000; Ti I–II 2228, III–IV 2126; Mt I–II 3034, III–IV 3036. Measurement of palps and legs: Pp 3.9 (1.0, 0.6, 0.8, –, 1.5); I 10.3 (3.1, 1.2, 2.6, 2.4, 1.0), II 13.3 (3.6, 1.5, 3.9, 2.9, 1.4), III 9.2 (2.9, 1.1, 2.0, 2.3, 0.9), IV 10.7 (3.4, 1.0, 2.4, 2.7, 1.2). Leg formula: II-IV-I-III. Chelicerae with three promarginal, four retromarginal teeth, and c. 22 denticles.

**Figure 6.**
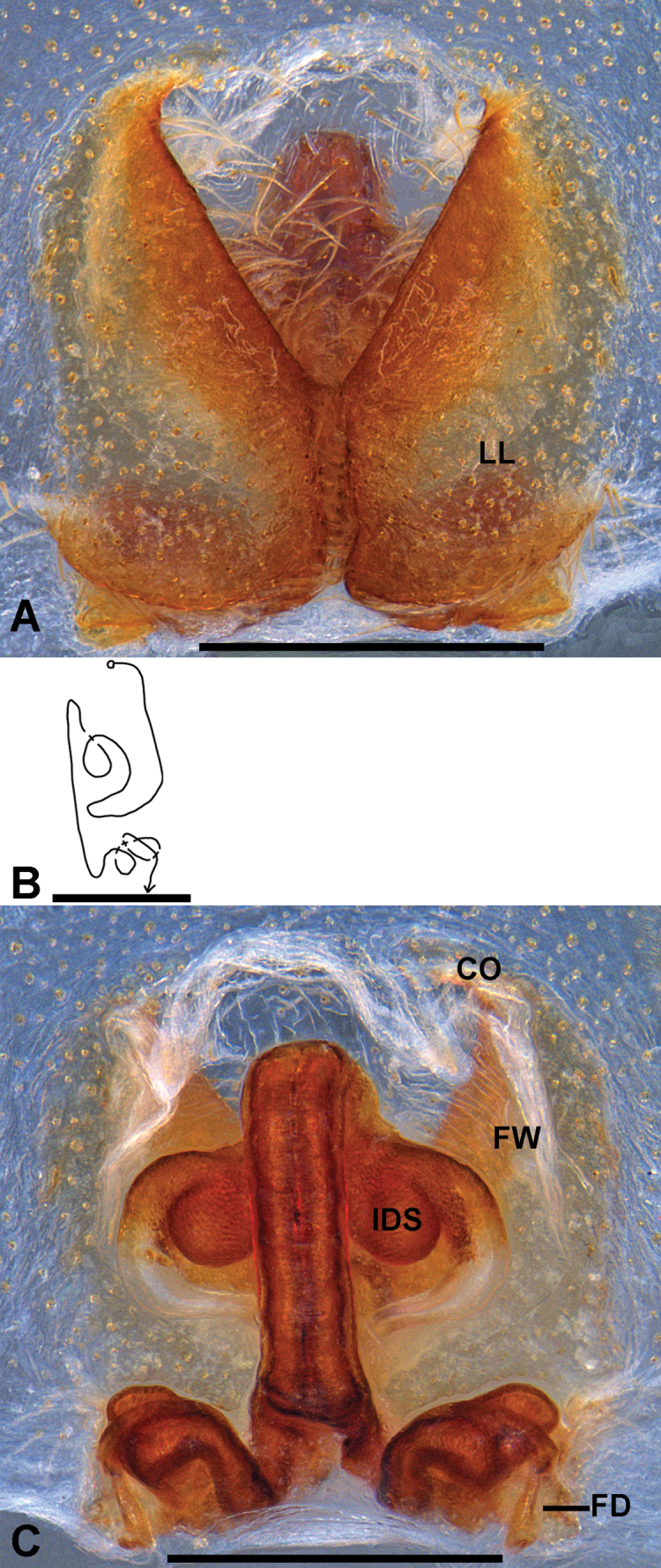
*
Pseudopoda spiralis* Zhang, Jäger & Liu, 2023 from Sichuan, female. A. Epigyne, ventral; B. Schematic course of IDS, dorsal; C. Vulva, dorsal. Abbreviations: CO, copulatory opening; FD, fertilization duct; FW, first winding; IDS, Internal duct system; LL, lateral lobes. Scale bars: 0.5 mm.

***Epigyne*** (Fig. 6A–C): As in diagnosis. Epigynal field as wide as long, without anterior bands. Anterior margins of lateral lobes forming a distinct V-shape, median margins of lateral lobes touching each other along the middle line. Internal duct system extends longitudinally and clearly visible in dorsal view, anterior margins slightly covered by FW. FD arising postero-laterally.

**Figure 7.**
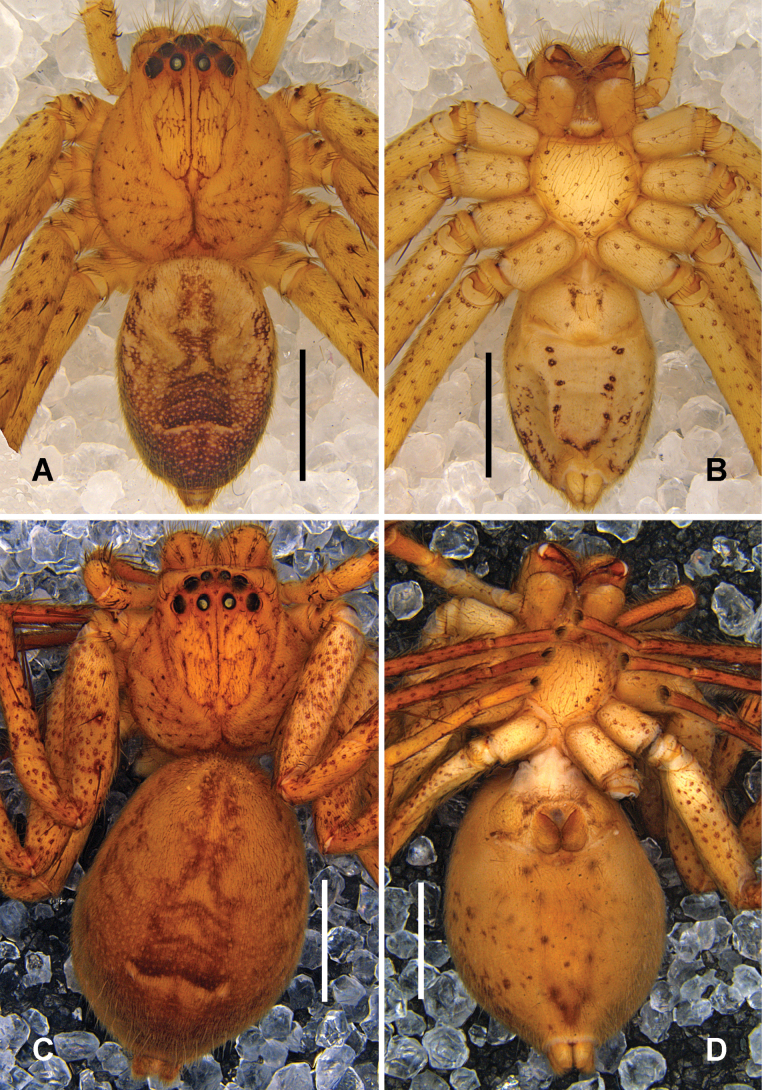
*
Pseudopoda spiralis* Zhang, Jäger & Liu, 2023 from Sichuan. A, B. Male habitus (A. Dorsal, B. Ventral); C, D. Female habitus (C. Dorsal, D. Ventral). Scale bars: 2 mm.

***Colouration*** (Fig. 7C, D): Carapace orange to reddish brown, with distinct radial streaks extending from the fovea to the margins. Eye region dark brown. Chelicerae orange to reddish brown; endites and labium orange-brown. Legs light orange, with numerous dark reddish-brown spots. OS dorsally yellowish brown with a darker median area and a transversal white line in posterior part. OS ventrally light yellow with irregular brown spots, denser laterally.

#### Distribution.

**Figure 8.**
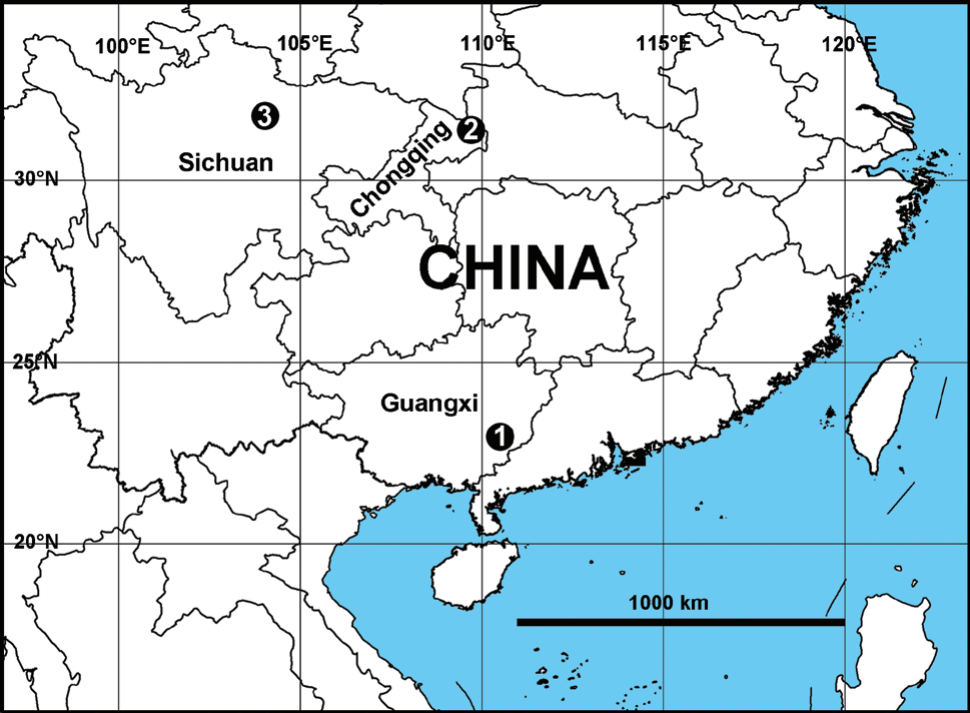
Distribution map of the three *Pseudopoda* species. The numbers represent the different species. 1. *P. rongxianensis* sp. nov.; 2. *P. deformis* Gong & Zhong, 2023; 3. *P. spiralis* Zhang, Jäger & Liu, 2023.

China (Sichuan Province, Fig. 8).

## Supplementary Material

XML Treatment for Pseudopoda

XML Treatment for Pseudopoda rongxianensis

XML Treatment for Pseudopoda deformis

XML Treatment for Pseudopoda spiralis

## ﻿Additional information

### Use of AI

No use of AI was reported.

### Author ORCIDs

He Zhang https://orcid.org/0000-0002-1478-9837

Chang-Hao Hu https://orcid.org/0009-0007-5591-3121

Lu-Yu Wang https://orcid.org/0000-0002-5250-3473

Jie Liu https://orcid.org/0000-0001-7744-9744

Yang Zhong https://orcid.org/0000-0002-0517-4582

### Data availability

All of the data that support the findings of this study are available in the main text.
